# Treatment Patterns and Economic Burden by Lines of Therapy Among Patients with Advanced Hepatocellular Carcinoma Treated with Systemic Cancer Therapy

**DOI:** 10.1007/s12029-019-00230-z

**Published:** 2019-04-23

**Authors:** Machaon M. Bonafede, Beata Korytowsky, Prianka Singh, Qian Cai, Katherine Cappell, Krutika Jariwala-Parikh, Bruce Sill, Neehar D. Parikh

**Affiliations:** 1 IBM Watson Health (formerly Truven Health Analytics Inc.), 75 Binney Street, Cambridge, MA 02142 USA; 2 Bristol-Myers Squibb, Princeton, NJ USA; 3 Department of Internal Medicine, University of Michigan, Ann Arbor, MI USA

**Keywords:** Advanced hepatocellular carcinoma, Treatment patterns, Sorafenib, Cost burden, Line of therapy, Survival

## Abstract

**Purpose:**

This study examined clinical and economic outcomes among patients with advanced hepatocellular carcinoma (HCC) treated with systemic agents by line of therapy.

**Methods:**

Adults with ≥ 2 medical claims for primary diagnosed HCC (from January 1, 2008, through September 30, 2015) and ≥ 1 claim for systemic HCC-related therapy were identified in the IBM MarketScan® Research Databases. Continuous enrollment was required 6 months before and 1 month after diagnosis. Patients were categorized into first- (1L) and second-line (2L) treatment cohorts; those receiving sorafenib as 1L were evaluated. Treatment patterns, healthcare resource utilization, costs, and survival during 1L and 2L therapy were measured. Survival was assessed for patients linked to the Social Security Administration Master Death File.

**Results:**

1459 patients, 758 with death data, met the 1L cohort criteria; 163 patients, 87 with death data, later received 2L therapy. 77.1% had 1L sorafenib, alone or in combination. Median 1L treatment duration was 3.0 months; median survival time from start of 1L to death or censor was 6.8 months. There was no predominant 2L agent. Median 2L treatment duration was 3.0 months; median survival time from start of 2L was 9.3 months. Median total healthcare costs per patient per month were $13,297 for 1L (all), $13,471 for 1L (sorafenib), and $11,786 for 2L.

**Conclusions:**

Findings confirm high 1-year mortality for advanced HCC, suggesting a high cost burden. While no 2L therapy was available during this analysis, recently approved 2L agents have the potential to improve survival after sorafenib failure or intolerance.

**Electronic supplementary material:**

The online version of this article (10.1007/s12029-019-00230-z) contains supplementary material, which is available to authorized users.

## Introduction

Hepatocellular carcinoma (HCC) is the most common primary liver cancer in the USA. The incidence of HCC tripled between 1975 and 2005 and has continued to increase, reaching an incidence of 6.7 cases per 100,000 persons in 2012 [1–4]. The leading risk factors for HCC are conditions that lead to liver cirrhosis, such as alcoholic liver disease, non-alcoholic steatohepatitis, or chronic infection with hepatitis B (HBV) or C virus (HCV) [1]. Patients diagnosed with early stage HCC who are eligible for surgical resection or transplantation can achieve 5-year survival rates of over 60%; therefore, there has been considerable focus on identification and monitoring of the at-risk population [2, 4]. However, for most patients who are diagnosed later with unresectable advanced HCC (aHCC) [3], 1-year survival is less than 40% and approved treatment options are limited [5, 6].

Sorafenib, an oral tyrosine kinase inhibitor with antiproliferative and antiangiogenic effects [7], was the first US Food and Drug Administration (FDA)-approved first-line (1L) therapy for the treatment of aHCC in 2007 [8]. Almost a decade later, in 2017, a second tyrosine kinase inhibitor, regorafenib, received FDA approval as a second-line (2L) therapy after disease progression on sorafenib [9, 10]. Because of similar mechanisms of action, regorafenib is restricted to patients who are able to tolerate sorafenib [11]. Nivolumab, an anti-programmed death-1 (PD-1) monoclonal antibody, was approved by the FDA in September 2017 as a 2L therapy for patients with aHCC after treatment with sorafenib [12]. More recently, lenvatinib, an oral tyrosine kinase inhibitor, was approved by the FDA for 1L therapy for patients with aHCC [13, 14]. Cabozantinib (a tyrosine kinase inhibitor) and pembrolizumab (a PD-1 blocking antibody) are additional 2L agents recently approved by the FDA for patients with aHCC previously treated with sorafenib [15, 16]. Regorafenib, ramucirumab, cabozantinib, nivolumab, and pembrolizumab are also recommended by the National Comprehensive Cancer Network guidelines for patients who progress on or after sorafenib [17].

Using the Surveillance, Epidemiology, and End Results (SEER)-Medicare linked database in 2009, the aggregate healthcare and lost productivity costs in the USA due to HCC was estimated to be $454.9 million annually, with $63.3 million attributed to aHCC [18]. It should be noted that although the SEER-Medicare dataset is comprehensive, it only includes cost data on patients over the age of 65 years with Medicare coverage, limiting the generalizability; therefore, cost estimates in younger patients are likely to be imprecise [19]. Other studies that were based on healthcare costs of HCC in general and aHCC specifically were limited by small sample size or utilized data that predate the approval of sorafenib [20, 21].

Given the limited evidence on pharmacological treatment patterns and economic outcomes for aHCC, we evaluated the real-world evidence on systemic therapy use, healthcare resource utilization, associated costs, and mortality by lines of therapy in the USA.

## Methods

### Study Design and Data Source

This retrospective cohort study used de-identified patient-level administrative claims data to analyze demographics, duration of therapy, healthcare resource utilization, costs, and mortality for patients with HCC on 1L and 2L systemic cancer therapy. Healthcare resource utilization, costs, and patient characteristics were extracted from the IBM MarketScan® Commercial and Medicare Supplemental Databases for the period between July 1, 2007, and March 31, 2016. The Commercial Database contains the pharmacy and medical (inpatient and outpatient) claims of employees and their dependents, and the Medicare Supplemental Database contains the healthcare claims of individuals with Medicare supplemental insurance paid for by employers. Both databases provide information about resource utilization and associated costs for healthcare services performed in both inpatient and outpatient settings. The MarketScan Research Databases comprises approximately 30 million patients annually covered by a geographically diverse group of self-insured employers and private insurance plans across the United States. The MarketScan Research Databases were further linked to the Social Security Administration Master Death File to obtain patient death events.

#### Patient Selection and Study Cohorts

Adult patients, aged ≥ 18 years, who had at least two non-diagnostic medical claims (30–180 days apart) with a primary HCC diagnosis (International Classification of Diseases, Ninth Revision, Clinical Modification [ICD-9-CM] 155.0x) between January 1, 2008, and September 30, 2015, were identified. The date of the earliest medical claim for HCC diagnosis was defined as the diagnosis index date. Patients were required to have continuous medical and pharmacy coverage for at least 6 months before and 30 days after the diagnosis index date. Patients with a diagnosis of primary or secondary cancer (including HCC) or with a claim for a systemic cancer agent before the diagnosis index date as well as those with a diagnosis of cholangiocarcinoma (ICD-9-CM 155.1×) were excluded from the study.

1L cohort

Eligible patients were included in the 1L cohort if they had at least one claim for an HCC-related systemic therapy, such as chemotherapy, targeted therapy, or immunotherapy, after the diagnosis index date. The 1L index date was defined as the date of the first claim for the 1L therapy. Patients were required to have continuous enrollment of 30 days after the 1L index date. A full list of HCC-related systemic therapies used in patient identification and analysis can be found in Electronic Supplementary Material 1. The subcohort of patients on 1L sorafenib was also identified and analyzed.

2L cohort

Patients from the 1L cohort were included in the 2L cohort if they had at least one claim for a non-1L systemic agent after the 1L index date. The 2L index date was defined as the date of the first claim for 2L therapy. To be included, patients had to have continuous enrollment from the diagnosis index date to 30 days after the 2L index date. Any HCC-related systemic therapies initiated within 60 days of the 1L and 2L index date were considered part of 1L/2L therapy. Some patients may have had more than one drug as a part of 1L/2L therapy if they had initiated more than one drug within 60 days of 1L/2L index date. Please see ‘Outcome Measures’ below for further detail on the definition of 1L and 2L therapy.

Patients on a 1L or 2L chemotherapy agent were excluded if they had an embolization (Current Procedural Terminology codes 37204, 75894, 36245, 36246, 36248, 75896, or 37243) within 30 days before or after the relevant index date. The intent of this criterion was to exclude patients who may have had an intratumoral infusion of chemotherapy treatment as part of transarterial chemoembolization and not as a systemic treatment.

### Baseline Characteristics

Patient demographics, including age, sex, geographic region (US census division), urban or rural residency, and type of insurance were reported. Urban or rural residence classification was based on whether the primary subscriber’s address was located within a Metropolitan Statistical Area. Recorded clinical characteristics included the Deyo–Charlson Comorbidity Index, an indicator of overall disease burden on the occurrence of at least one of 17 comorbid conditions identified using the ICD-9-CM coding manual. In addition to the Deyo–Charlson Comorbidity Index, general comorbid conditions (anxiety, cardiovascular disease, chronic obstructive pulmonary disease/asthma, depression, hypertension, osteoarthritis, and osteoporosis), liver-related conditions (non-alcoholic steatohepatitis, alcoholic liver disease, HBV status, and HCV status), and cirrhosis status were also assessed.

Demographic characteristics were reported on the cohort-specific index date (1L or 2L index date). Clinical characteristics were measured during the 6-month period before the cohort-specific index date.

### Outcome Measures

The duration of therapy was assessed for 1L and 2L, calculated from the 1L and 2L index dates to the earliest of the following: death, end of continuous enrollment, end of data (March 31, 2016), discontinuation, switch, or augmentation. Discontinuation was defined as a gap of ≥ 60 days in medication supply, switching was defined as initiation of a new systemic cancer therapy without continued use of the index therapy > 60 days after the 1L or 2L index date, and augmentation was defined as the initiation of a new systemic cancer therapy with continued use of the index therapy > 60 days after the 1L or 2L index date. In addition to duration of therapy, time to initiation of 1L/2L therapy, reason for terminating a line of therapy, and type of systemic drug(s) at 1L and 2L index were recorded.

All-cause healthcare resource utilization—including inpatient admissions, emergency room visits, and outpatient services such as physician office visits, laboratory tests, and radiology exams—was recorded for each cohort (1L all, 1L sorafenib, and 2L all), for duration of therapy. For patients in the 1L sorafenib cohort, the time to first inpatient admission and the treatment quartile of each inpatient admission were recorded to determine the timing of inpatient stays during the course of treatment. Treatment quartiles were determined on a per-patient basis as one-quarter of a patient’s duration of therapy.

All-cause total healthcare costs, including medical services costs (inpatient admissions, emergency room visits, and outpatient services) and outpatient pharmacy costs over the duration of therapy, were reported for the 1L and 2L cohorts. Total costs were defined as the sum of health plan and patient-paid costs incurred from fully adjudicated medical and pharmacy claims. All costs were reported as per patient per month and adjusted to 2015 US dollars using the medical care component of the Consumer Price Index [22]. End of follow-up for healthcare utilization and cost outcomes was the earliest instance of inpatient death or Social Security Administration death, end of continuous enrollment, or end of study (i.e., March 31, 2016).

To evaluate survival outcomes, the analysis was limited to the subset of patients in the commercial and Medicare databases that could be linked to the Social Security Administration death data. Among the subset of linked patients, we assessed whether they experienced death or not. Date of death was determined based on the death record from the linked Social Security Administration Master Death File. Survival time was calculated as the time from the 1L and 2L index date to the death date (if the patient died) or censor date (end of continuous enrollment or study period), whichever occurred first.

#### Statistical Analysis

For all study variables, mean, standard deviation, and median were reported for all continuous variables, and frequencies and percentages were reported for categorical variables. All data analyses were conducted using SAS version 9.4 (SAS Inc., Cary, NC, USA). Data availability: Bristol-Myers Squibb policy on data sharing may be found at https://www.bms.com/researchers-and-partners/independent-research/data-sharing-request-process.html.

## Results

### Demographic and Clinical Characteristics

**Fig. 1:**
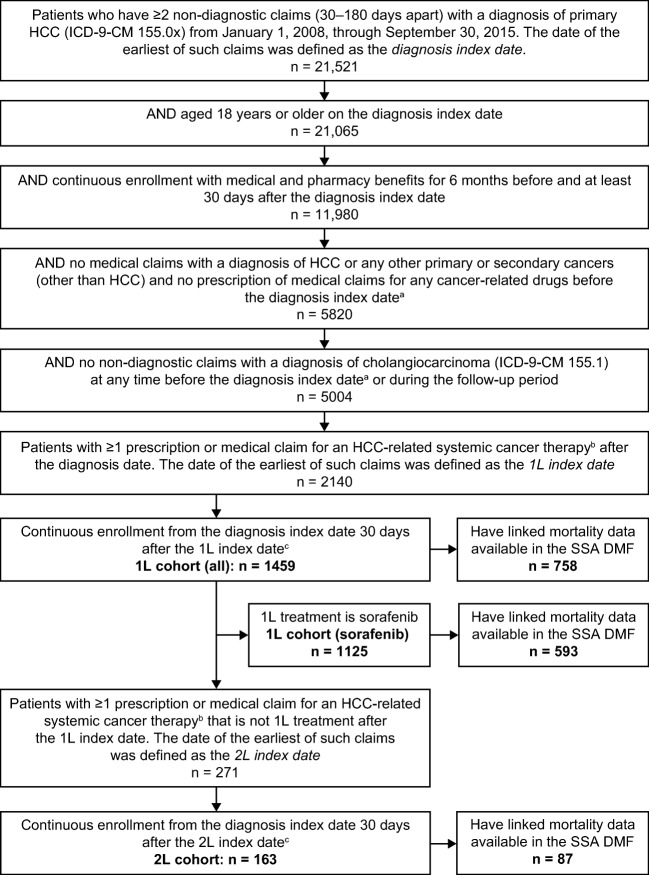
Patient selection. *ICD-9-CM* International Classification of Diseases, ninth revision, clinical modification, *SSA DMF* Social Security Administration Death Master File. Superscript a, the look-back period began on January 1, 2005 (or start of database enrollment, whichever is later), and ended on the day before the index date; superscript b, for list see Electronic Supplementary Material 1; superscript c, excludes patients with 1L chemotherapy who had an embolization within ± 30 days of 1L index date

A total of 1459 patients met the inclusion criteria for the 1L cohort. Of these patients, 163 (11.2%) met the criteria for inclusion in the 2L cohort (Fig. 1). The mean age of patients in the 1L cohort was 61.7 ± 10.1 years and the majority were males (78.1%). Most patients (77.1%) were treated with 1L sorafenib; the mean age of this subcohort was 62.0 ± 10.1 years and 81.4% were male. The mean age of the 2L cohort was 61.0 ± 9.1 years and 74.2% were males (Table 1). Over 40% of patients from all cohorts were from the US South Census region and more than 85% were urban residents. The majority had health insurance coverage through exclusive provider organizations or preferred provider organizations (Table 1).

**Table 1:** Demographic and clinical characteristics

	First-line cohort (all)		First-line cohort (sorafenib)		Second-line cohort (all)	
	*N* = 1459		*N* = 1125		*N* = 163	
Demographics						
Age (mean, SD), years	61.7	10.1	62.0	10.1	61.0	9.1
Male (*N*, %)	1140	78.1%	916	81.4%	121	74.2%
Geographic region (*N*, %)						
Northeast	260	17.8%	204	18.1%	36	22.1%
North Central	288	19.7%	211	18.8%	31	19.0%
South	593	40.6%	454	40.4%	69	42.3%
West	290	19.9%	236	21.0%	25	15.3%
Unknown	28	1.9%	20	1.8%	2	1.2%
Insurance plan type (*N*, %)						
Comprehensive/indemnity	218	14.9%	180	16.0%	23	14.1%
EPO/PPO	774	53.1%	587	52.2%	85	52.2%
POS/POS with capitation	85	5.8%	71	6.3%	11	6.8%
HMO	252	17.3%	198	17.6%	25	15.3%
CDHP/HDHP	73	5.0%	50	4.4%	13	8.0%
Unknown	57	3.9%	39	3.5%	6	3.7%
Rural residence indicator (*N*, %)						
Urban	1250	85.7%	976	86.8%	140	85.9%
Rural	182	12.5%	129	11.5%	21	12.9%
Unknown	27	1.9%	20	1.8%	2	1.2%
Clinical characteristics						
Deyo–Charlson Comorbidity Index (mean, SD)	5.7	3.1	5.5	3.1	6.3	3.3
Comorbid conditions (*N*, %)						
Anxiety	59	4.0%	44	3.9%	8	4.9%
Cardiovascular disease	488	33.5%	375	33.3%	46	28.2%
Chronic obstructive pulmonary disease/asthma	141	9.7%	115	10.2%	13	8.0%
Depression	72	4.9%	52	4.6%	14	8.6%
Diabetes	491	33.7%	397	35.3%	44	27.0%
Hypertension	608	41.7%	461	41.0%	59	36.2%
Osteoarthritis	89	6.1%	72	6.4%	5	3.1%
Osteoporosis	11	0.8%	5	0.4%	1	0.6%
Liver-related comorbidities (*N*, %)						
Non-alcoholic steatohepatitis	76	5.2%	59	5.2%	7	4.3%
Alcoholic liver disease	167	11.5%	141	12.5%	8	4.9%
Hepatitis B virus only	84	5.8%	74	6.6%	3	1.8%
Hepatitis C virus only	554	38.0%	458	40.7%	58	35.6%
Hepatitis B and C virus	35	2.4%	26	2.3%	3	1.8%
Cirrhosis status	646	44.3%	543	48.3%	44	27.0%

*CDHP*, consumer-driven health plan; *EPO*, exclusive provider organization; *HDHP*, high-deductible health plan; *HMO*, health maintenance organization; *POS*, point of service; *PPO*, preferred provider organization; *SD*, standard deviation

The mean baseline Deyo–Charlson Comorbidity Index of patients in the 1L (all), 1L (sorafenib), and 2L cohorts was 5.7 ± 3.1, 5.5 ± 3.1, and 6.3 ± 3.3, respectively. The top three comorbid conditions at baseline were hypertension (1L-all, 41.7%; 1L-sorafenib, 41.0%; 2L, 36.2%), diabetes (1L-all, 33.7%; 1L-sorafenib, 35.3%; 2L, 27.0%), and cardiovascular disease (1L-all, 33.5%; 1L-sorafenib, 33.3%; 2L, 28.2%). The frequency of cirrhosis was much higher than that of alcoholic liver disease in the pre-index period for all three cohorts: 44.3% versus 11.5% in the 1L (all), 48.3% versus 12.5% in 1L (sorafenib) subcohort, and 27% versus 4.9% in the 2L cohort. Among the 1L (all) cohort, 38% of patients had HCV only, 5.8% had HBV only, and 2.4% had both HCV and HBV; a similar distribution was found in the 1L (sorafenib) subcohort and the 2L cohort.

#### Treatment Patterns

The treatment patterns for the 1L and 2L cohorts are shown in Table 2. In brief, the mean (median) time from diagnosis index date to 1L index date was 5.24 ± 8.93 (1.63) months. For patients who progressed to 2L treatment, the mean (median) time from the end of 1L therapy to the 2L index date was 3.06 ± 5.39 (1.07) months. Sorafenib monotherapy was the most commonly prescribed 1L therapy and the mean time to treatment initiation for this subcohort was 4.17 ± 7.02 months. For 2L therapies, the most common choices were systemic chemotherapy (*n* = 80; 49.1%) and targeted therapy other than sorafenib (*n* = 70; 42.9%). Across the patient population, a total of 68 different 1L and 44 different 2L treatment regimens, including combinations, were identified. The top five regimens used by patients in the 1L cohort included sorafenib (74%); sirolimus (6%); everolimus (2%); gemcitabine (2%); and doxorubicin and sorafenib combination (2%) (see Electronic Supplementary Material 2). The top five regimens used by patients in the 2L cohort included sirolimus (17%); fluorouracil (8%); sorafenib (7%); capecitabine (7%); everolimus (6%); and gemcitabine (6%) (see Electronic Supplementary Material 3).

**Table 2:** Treatment patterns

	First-line cohort (all)		First-line cohort (sorafenib)		Second-line cohort (all)	
	*N* = 1459		*N* = 1125		*N* = 163	
Months to treatment start (mean, SD)	5.24	8.93	4.17	7.02	3.06	5.39
Months to start (median)	1.63		1.43		1.07	
Index therapy^a,b^ (*N*, %)						
Systemic chemotherapy	181	12.4%	1	0.1%	80	49.1%
Targeted therapies without sorafenib	165	11.3%	0	0.0%	70	42.9%
Immunotherapy	1	0.1%	0	0.0%	4	2.5%
Sorafenib	1125	77.1%	1125	100.0%	15	9.2%
Reason for termination (*N*, %)						
Discontinuation	609	41.7%	451	40.1%	70	42.9%
Switching	81	5.6%	54	4.8%	17	10.4%
Augmentation	29	2.0%	15	1.3%	8	4.9%
Death	190	13.0%	169	15.0%	12	7.4%
End of enrollment	515	35.3%	421	37.4%	44	27.0%
End of study	35	2.4%	15	1.3%	12	7.4%
Duration of therapy, months (mean, SD)	5.06	5.98	4.90	5.63	5.13	7.29
Duration of therapy, months (median)	3.03		3.00		3.03	
Duration of 2L sorafenib, months (mean, SD)					6.82	4.06
Duration of 2L sorafenib, months (median)					7.47	

*2L*, second line; *SD*, standard deviation

^a^Patients can receive more than one type of medication on the index date

^b^See Online Resource Material 1 for a full list of therapies

The mean duration of therapy was 5.06 ± 5.98 months with a median of 3.03 months in the 1L (all) cohort, 4.90 ± 5.63 months with a median of 3.00 months in the 1L (sorafenib) subcohort, and 5.13 ± 7.29 months with a median of 3.03 months in the 2L cohort.

#### Healthcare Resource Utilization and Costs

**Fig. 2:**
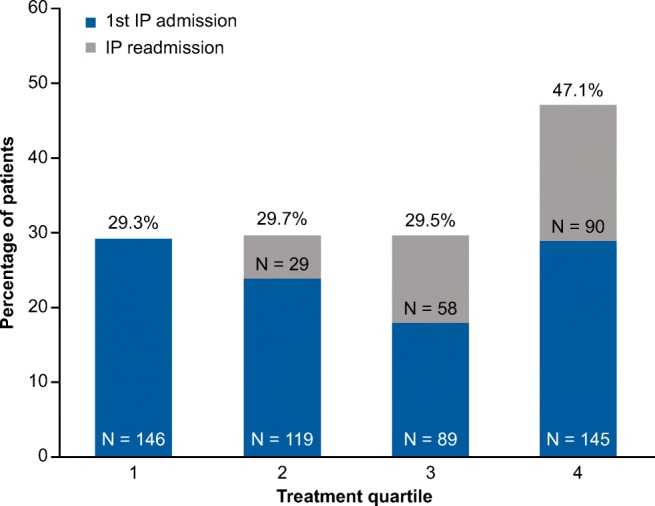
Percentage of first-line sorafenib patients with at least one IP admission (*N* = 499) whose IP admission occurred within each treatment quartile. *IP* inpatient

During 1L therapy, 42.9% of patients in the 1L cohort had at least one inpatient admission, and 38.6% had at least one emergency room visit (Table 3). Of the 499 patients in the 1L sorafenib subcohort with at least one inpatient admission, 53% had their first inpatient admission during the first half of their therapy (Fig. 2). The number of inpatient readmissions increased steadily through the fourth treatment quartile.

**Table 3:** All-cause healthcare resource utilization and costs

	First-line cohort (all)			First-line cohort (sorafenib)			Second-line cohort (all)		
	*N* = 1459			*N* = 1125			*N* = 163		
	*N*/mean	%/SD	Median	*N*/mean	%/SD	Median	*N*/mean	%/SD	Median
Medical services									
IP admissions									
Patients with any IP admission (*N*, %)	626	42.9%		499	44.4%		55	33.7%	
No. of IP admissions, PPPM^a^ (mean, SD)	0.2	0.4		0.2	0.4		0.2	0.4	
IP costs, PPPM (mean, SD, median)	$5605	$14,887	$0	$5705	$12,561	$0	$4635	$23,174	$0
ER visits									
Patients with any ER visit (*N*, %)	563	38.6%		451	40.1%		33	20.3%	
No. of ER visits, PPPM (mean, SD)	0.3	0.7		0.4	0.8		0.3	1.6	
ER costs, PPPM (mean, SD, median)	$246	$1727	$0	$206	$808	$0	$121	$481	$0
OP services									
Total OP costs, PPPM (mean, SD, median)	$6118	$11,517	$2723	$5067	$11,681	$2144	$11,194	$18,156	$5761
Physician office visits									
No. of office visits, PPPM (mean, SD)	2.2	1.6		2.1	1.5		2.4	2.1	
Office visit costs, PPPM (mean, SD, median)	$303	$506	$217	$305	$533	$215	$355	$840	$210
Laboratory services									
No. of laboratory services, PPPM (mean, SD)	2.2	2.0		1.9	1.7		2.8	2.4	
Laboratory costs, PPPM (mean, SD, median)	$401	$749	$138	$328	$645	$112	$419	$639	$173
Radiology services									
No. of radiology services, PPPM (mean, SD)	1.5	2.4		1.4	2.1		2.0	3.9	
Radiology costs, PPPM (mean, SD, median)	$1187	$3414	$289	$1134	$3512	$240	$2187	$8981	$414
Other OP services									
No. of other OP services, PPPM (mean, SD)	4.3	5.1		3.6	3.9		7.2	8.8	
Other OP service costs, PPPM (mean, SD, median)	$4227	$10,195	$1286	$3300	$10,326	$900	$8232	$14,631	$4037
OP pharmacy									
No. of OP pharmacy claims, PPPM (mean, SD)	5.3	5.5		5.0	3.0		5.2	3.4	
OP pharmacy costs, PPPM (mean, SD, median)	$6413	$4728	$6245	$7581	$4006	$7514	$3609	$5252	$1271
Total medical costs^b^, PPPM (mean, SD, median)	$11,968	$19,130	$5879	$10,978	$17,438	$4973	$15,950	$30,179	$8142
Total healthcare costs^c^, PPPM (mean, SD, median)	$18,381	$19,633	$13,297	$18,559	$18,012	$13,471	$19,559	$30,065	$11,786

*ER*, emergency room; *IP*, inpatient; *OP*, outpatient; *PPPM*, per-patient-per-month; *SD*, standard deviation

^a^Counts and expenditures are reported PPPM due to the variable follow-up period

^b^Medical costs include IP admission costs, ER visit costs, and OP services costs

^c^Healthcare costs include medical costs and OP pharmacy costs

Mean all-cause per-patient per-month total healthcare costs for 1L therapy were $18,381 ± $19,633 and median costs were $13,297. In the 1L sorafenib subcohort, 40.1% had at least one emergency room visit (Table 3). Mean all-cause per-patient per-month total healthcare costs for this subcohort were $18,559 ± $18,012 and median costs were $13,471.

During 2L therapy, 33.7% of patients had at least one inpatient admission, and 20.3% had at least one emergency room visit (Table 3). Mean all-cause per-patient per-month total healthcare costs for 2L therapy were $19,559 ± $30,065 and median costs were $11,786. Medical costs represented 65% of 1L (all) healthcare costs, 59% of 1L (sorafenib) healthcare costs, and 82% of 2L healthcare costs. Additional detail on healthcare resource utilization and costs for all cohorts can be found in Table 3.

### Survival Outcomes

More than half of the study patients could be linked to the Social Security Administration Master Death File (*n* = 758 for 1L [all] cohort; *n* = 593 for 1L [sorafenib] subcohort; *n* = 87 for 2L cohort), enabling the assessment of survival outcomes. Of them, more than half died during the follow-up period (54.0% of the 1L [all], 58.2% of the 1L [sorafenib], and 52.9% of the 2L cohort; Table 4). Median survival time, from cohort 1L and 2L index date to death or censor, was 6.8 months for 1L (all) patients, 6.0 months for 1L (sorafenib) patients, and 9.3 months for 2L patients. One-year survival was 32.6% for 1L (all) therapy, 28.5% for 1L (sorafenib), and 34.5% for 2L therapy. Survival rates at 3 years were 6.6% for 1L (all) therapy, 5.4% for 1L sorafenib, and 5.8% for 2L therapy.

**Table 4:** Survival during the follow-up period

	First-line cohort (all)		First-line cohort (sorafenib)		Second-line cohort (all)	
	*N* = 758		*N* = 593		*N* = 87	
Died (*N*, %)	409	54.0%	345	58.2%	46	52.9%
Survival time, months^a^ (mean, SD)	12.28	14.34	11.09	13.13	13.78	14.14
Survival time, months^a^ (median)	6.83		5.97		9.33	
Patients who survived^b^ (*N*, %)						
< 1 year	511	67.4%	424	71.5%	57	65.5%
≥ 1	247	32.6%	169	28.5%	30	34.5%
≥ 2	94	12.4%	66	11.1%	14	16.1%
≥ 3	50	6.6%	32	5.4%	5	5.7%
≥ 4	30	4.0%	18	3.0%	4	4.6%

*SD* standard deviation

^a^Time from cohort index date to death date or censor date (end of MarketScan enrollment or end of study period)

^b^Counts are not mutually exclusive

## Discussion

This retrospective claims study is the first real-world analysis that presents comprehensive data on treatment patterns, healthcare resource utilization, healthcare costs, and survival outcomes by line of therapy in patients with aHCC who received systemic cancer therapy. Our findings showed that the overall survival outcomes were poor in both lines of therapy with high healthcare resource utilization and economic burden in both the 1L and 2L setting and in the sorafenib 1L subcohort; only a low percentage of patients progressed to 2L due to the burden of HCC and the lack of standardized treatment options. Sorafenib has been the standard of care in 1L aHCC since its approval in 2005. However, there remains an unmet need for 2L and 1L alternatives to sorafenib that improve outcomes with fewer side effects while maintaining quality of life and cost savings.

In this high-cost population, medical expenses—consisting of inpatient hospitalization and outpatient services costs—exceeded $10,000 ($4500) per patient per month for both the 1L and 2L cohorts and in the 1L sorafenib subcohort. In particular, the strong positive skew of cost data was driven by high rates of inpatient admissions; therefore, medications and management strategies that are both effective and minimize hospitalizations due to adverse events or disease-related complications are needed to maximize value [20]. Our results on the cost of systemic therapy for aHCC are consistent with previous reports in which the estimated monthly healthcare costs of patients receiving 1L sorafenib have ranged from $6000 to over $16,000 [20, 23].

Given the trend towards value-based care [24], there is a need to have therapies evaluated for cost-effectiveness, a measure that incorporates both economic and clinical outcomes of the drug [25]. Recently, the tyrosine kinase inhibitor lenvatinib was approved for 1L treatment of aHCC [13, 14], while the multikinase inhibitors regorafenib and cabozantinib and the immuno-oncology drugs nivolumab and pembrolizumab were approved as 2L therapies [9, 10, 12, 26].

As the treatment landscape of aHCC shifts with the entry of newer agents, cancer-specific patient-reported outcome measures will be essential to capture differences in patient quality of life in cases where standard outcome metrics demonstrate equivalency between treatments [24, 27, 28]. For example, a recent review of patient-reported outcomes associated with nivolumab treatment of advanced solid cancers reported that, in addition to clinical benefits, nivolumab was associated with stabilization or improvement of patient quality of life [28]. This is in contrast to chemotherapy agents and targeted therapies that are generally associated with deterioration in quality of life.

### Limitations

The limitations of this study are similar to those seen in other claims-based observational studies. First, this study was restricted to those individuals with commercial health coverage or private Medicare supplemental coverage; therefore, results may not be generalizable to patients with other insurance types or without health insurance coverage. This population is younger than the general aHCC population and may overestimate the efficacy and duration of treatment and health-related outcomes. Second, claim data are collected for administrative purposes; therefore, the data are subject to coding limitations and data entry errors, lack indicators of clinical status such as Eastern Cooperative Oncology Group scores or cancer staging, and only reflect direct healthcare costs via the paid amounts of adjudicated claims to individual hospitals and providers. Third, death data were only available for the subset of individuals who could be linked to the Social Security Administration Master Death File. Fourth, patients with aHCC who did not receive systemic cancer therapy, such as those who received only palliative care, were not evaluated in this study, and misclassification bias due to the implementation of line of therapy algorithms may occur in this study. Lastly, the study was completed before the approval of current 2L therapies for aHCC, so the treatment landscape may look different in future analyses. However, given the recentness of this evolution in care, this study provides a baseline for survival, healthcare utilization and cost prior to the approval of these new agents.

## Conclusions

In this real-world claims analysis of patients with aHCC, most patients received sorafenib as 1L treatment; however, 23% of patients were treated with non-approved 1L therapies and there were no apparent trends in the selection of 2L therapies. Healthcare costs were substantial, primarily driven by pharmacy costs for the 1L cohort and by outpatient services for the 2L cohort. The high 1-year mortality rate and economic burden associated with aHCC underscores the continued need for more effective pharmacologic treatments for this patient population.

## Electronic Supplementary Material

ESM 1 (PDF 128 kb)

ESM 2 (PDF 38 kb)

ESM 3 (PDF 29 kb)
