# Supplementary material for: Treatment Patterns and Economic Burden by Lines of Therapy Among Patients with Advanced Hepatocellular Carcinoma Treated with Systemic Cancer Therapy
Source: J Gastrointest Cancer. 2019 Apr 23;51(1):217–26. doi: 10.1007/s12029-019-00230-z (PMC7000498; doi:10.1007/s12029-019-00230-z)
Supplement: Supplementary file 1 — (PDF 128 kb) [file 12029_2019_230_MOESM1_ESM.pdf]

Treatment Patterns and Economic Burden by Lines of Therapy among Patients with Advanced Hepatocellular Carcinoma Treated with Systemic Cancer Therapy

Journal of Gastrointestinal Cancer

Machaon M. Bonafede, Beata Korytowsky, Prianka Singh, Qian Cai, Katherine Cappell, Krutika Jariwala-Parikh, Bruce Sill, Neehar D. Parikh

Corresponding Author: Machaon M. Bonafede, PhD, MPH
Affiliation: IBM Watson Health
Address: 75 Binney Street, Cambridge, MA 02142
E-mail: mbonafed@us.ibm.com

Electronic Supplementary Material 1 Hepatocellular cancer-related systemic cancer agents

Treatment Type	Generic Drug Names
Systemic chemotherapy	Capecitabine, carboplatin, cisplatin, doxorubicin, 5-fluororacil, gemcitabine, vincristine, irinotecan
Targeted therapy	Axitinib, bevacizumab, bortezomib, cabozantinib, cetuximab, crizotinib, erlotinib, everolimus, gefitinib, lenvatinib, nintedanib, pazopanib, ramucirumab, regorafenib, sirolimus, sorafenib, sunitinib, temsirolimus
Immunotherapy	Ipilimumab, nivolumab, pembrolizumab
