# Supplementary material for: Treatment Patterns and Economic Burden by Lines of Therapy Among Patients with Advanced Hepatocellular Carcinoma Treated with Systemic Cancer Therapy
Source: J Gastrointest Cancer. 2019 Apr 23;51(1):217–26. doi: 10.1007/s12029-019-00230-z (PMC7000498; doi:10.1007/s12029-019-00230-z)
Supplement: Supplementary file 2 — (PDF 38 kb) [file 12029_2019_230_MOESM2_ESM.pdf]

Treatment Patterns and Economic Burden by Lines of Therapy among Patients with Advanced Hepatocellular Carcinoma Treated with Systemic Cancer Therapy

Journal of Gastrointestinal Cancer

Machaon M. Bonafede, Beata Korytowsky, Prianka Singh, Qian Cai, Katherine Cappell, Krutika Jariwala-Parikh, Bruce Sill, Neehar D. Parikh

Corresponding Author: Machaon M. Bonafede, PhD, MPH
Affiliation: IBM Watson Health
Address: 75 Binney Street, Cambridge, MA 02142
E-mail: mbonafed@us.ibm.com

Electronic Supplementary Material 2 1L treatment regimens (*N* = 1459)

**First-line therapy**	**Patients (*N*)**	**%**
bevacizumab	11	0.8%
bevacizumab capecitabine	1	0.1%
bevacizumab capecitabine 5-fluorouracil	1	0.1%
bevacizumab carboplatin	2	0.1%
bevacizumab carboplatin 5-fluorouracil	1	0.1%
bevacizumab carboplatin gemcitabine	1	0.1%
bevacizumab carboplatin sorafenib	1	0.1%
bevacizumab cisplatin	1	0.1%
bevacizumab cisplatin gemcitabine	1	0.1%
bevacizumab 5-fluorouracil	10	0.7%
bevacizumab 5-fluorouracil irinotecan	1	0.1%
bevacizumab gemcitabine sorafenib	1	0.1%
bevacizumab sorafenib	1	0.1%
bortezomib	1	0.1%
capecitabine	16	1.1%
capecitabine cisplatin	1	0.1%
capecitabine cisplatin gemcitabine	1	0.1%
capecitabine cisplatin sorafenib	1	0.1%
capecitabine gemcitabine	3	0.2%
capecitabine sorafenib	3	0.2%
carboplatin	22	1.5%
carboplatin cisplatin	2	0.1%
carboplatin cisplatin gemcitabine	1	0.1%
carboplatin doxorubicin	1	0.1%
carboplatin erlotinib gemcitabine	1	0.1%
carboplatin 5-fluorouracil gemcitabine	1	0.1%
carboplatin gemcitabine	5	0.3%
carboplatin gemcitabine sirolimus sorafenib	1	0.1%
carboplatin gemcitabine sorafenib	1	0.1%
carboplatin sorafenib	1	0.1%
cetuximab gemcitabine	2	0.1%
cetuximab irinotecan	1	0.1%
cisplatin	12	0.8%
cisplatin doxorubicin	1	0.1%
cisplatin doxorubicin 5-fluorouracil	5	0.3%
cisplatin doxorubicin gemcitabine	1	0.1%
cisplatin doxorubicin sorafenib	3	0.2%
cisplatin erlotinib gemcitabine	1	0.1%
cisplatin 5-fluorouracil	1	0.1%

cisplatin<br>5-fluorouracil gemcitabine	1	0.1%
cisplatin<br>5-fluorouracil irinotecan	1	0.1%
cisplatin<br>5-fluorouracil vincristine	1	0.1%
cisplatin gemcitabine	10	0.7%
cisplatin gemcitabine sorafenib	1	0.1%
doxorubicin	12	0.8%
doxorubicin sirolimus	1	0.1%
doxorubicin sorafenib	28	1.9%
doxorubicin vincristine	5	0.3%
erlotinib	1	0.1%
erlotinib gemcitabine	1	0.1%
erlotinib sorafenib	1	0.1%
enviroximes	30	2.1%
everolimus sirolimus	1	0.1%
everolimus sorafenib	1	0.1%
5-fluorouracil	14	1.0%
5-fluorouracil gemcitabine	1	0.1%
5-fluorouracil irinotecan	5	0.3%
5-fluorouracil sorafenib	2	0.1%
gemcitabine	30	2.1%
gemcitabine sorafenib	1	0.1%
ipilimumab	1	0.1%
regorafenib	1	0.1%
sirolimus	85	5.8%
sirolimus sorafenib	11	0.8%
sorafenib	1080	74.0%
sorafenib sunitinib	3	0.2%
sunitinib	7	0.5%
vincristine	1	0.1%
**Total**	**1459**	100.0%
