# Supplementary material for: Treatment Patterns and Economic Burden by Lines of Therapy Among Patients with Advanced Hepatocellular Carcinoma Treated with Systemic Cancer Therapy
Source: J Gastrointest Cancer. 2019 Apr 23;51(1):217–26. doi: 10.1007/s12029-019-00230-z (PMC7000498; doi:10.1007/s12029-019-00230-z)
Supplement: Supplementary file 3 — (PDF 29 kb) [file 12029_2019_230_MOESM3_ESM.pdf]

**Treatment Patterns and Economic Burden by Lines of Therapy among Patients with Advanced Hepatocellular Carcinoma Treated with Systemic Cancer Therapy**

*Journal of Gastrointestinal Cancer*

Machaon M. Bonafede, Beata Korytowsky, Prianka Singh, Qian Cai, Katherine Cappell, Krutika Jariwala-Parikh, Bruce Sill, Neehar D. Parikh

**Corresponding Author:** Machaon M. Bonafede, PhD, MPH
**Affiliation:** IBM Watson Health
**Address:** 75 Binney Street, Cambridge, MA 02142
**E-mail:** mbonafed@us.ibm.com

**Electronic Supplementary Material 3** 2L treatment regimens (*N* = 163)

| Second-line therapy | Patients (*N*) | % |
|---|---|---|
| bevacizumab | 4 | 2.5% |
| bevacizumab capecitabine | 1 | 0.6% |
| bevacizumab capecitabine 5-fluorouracil sorafenib | 1 | 0.6% |
| bevacizumab doxorubicin gemcitabine | 1 | 0.6% |
| bevacizumab erlotinib | 2 | 1.2% |
| bevacizumab erlotinib sorafenib | 1 | 0.6% |
| bevacizumab 5-fluorouracil | 1 | 0.6% |
| bevacizumab 5-fluorouracil irinotecan | 3 | 1.8% |
| bevacizumab gemcitabine | 1 | 0.6% |
| bevacizumab irinotecan | 1 | 0.6% |
| bevacizumab sorafenib | 1 | 0.6% |
| capecitabine | 11 | 6.7% |
| carboplatin | 6 | 3.7% |
| carboplatin doxorubicin gemcitabine | 1 | 0.6% |
| cetuximab irinotecan | 1 | 0.6% |
| cisplatin | 1 | 0.6% |
| cisplatin gemcitabine | 4 | 2.5% |
| cisplatin irinotecan | 2 | 1.2% |
| doxorubicin | 7 | 4.3% |
| doxorubicin 5-fluorouracil sorafenib | 1 | 0.6% |
| doxorubicin sirolimus | 1 | 0.6% |
| doxorubicin sorafenib | 5 | 3.1% |
| erlotinib | 6 | 3.7% |
| enviroximes | 10 | 6.1% |
| everolimus sirolimus | 1 | 0.6% |
| everolimus sirolimus sorafenib | 1 | 0.6% |
| everolimus sorafenib | 1 | 0.6% |
| 5-fluorouracil | 13 | 8.0% |
| 5-fluorouracil irinotecan | 4 | 2.5% |
| 5-fluorouracil sirolimus | 2 | 1.2% |
| gemcitabine | 10 | 6.1% |
| gemcitabine nivolumab | 1 | 0.6% |
| gemcitabine sunitinib | 1 | 0.6% |
| ipilimumab | 1 | 0.6% |
| irinotecan | 1 | 0.6% |
| nivolumab | 2 | 1.2% |
| pazopanib | 2 | 1.2% |
| regorafenib sirolimus | 1 | 0.6% |
| sirolimus | 27 | 16.6% |
| sirolimus sorafenib | 4 | 2.5% |

| | | |
|---|---|---|
| sorafenib | 12 | 7.4% |
| sorafenib sunitinib | 1 | 0.6% |
| sunitinib | 4 | 2.5% |
| vincristine | 1 | 0.6% |
| **Total** | 163 | 100.0% |
